# Electrochemical Characterization of Protein Adsorption onto YNGRT-Au and VLGXE-Au Surfaces

**DOI:** 10.3390/s150819429

**Published:** 2015-08-07

**Authors:** Hanna Trzeciakiewicz, Jose Esteves-Villanueva, Rania Soudy, Kamaljit Kaur, Sanela Martic-Milne

**Affiliations:** 1 Department of Chemistry, Oakland University, 2200 North Squirrel Road, Rochester, MI 48309, USA; E-Mails: htrzecia@oakland.edu (H.T.); joesteve@oakland.edu (J.E.-V.); 2 Faculty of Pharmacy and Pharmaceutical Sciences, University of Alberta, 87 Avenue, Edmonton, AB T6G 2E1, Canada; E-Mails: soudy@ualberta.ca (R.S.); kkaur@chapman.edu (K.K.); 3 Chapman University School of Pharmacy, Harry and Diane Rinker Health Science Campus, Chapman University, Irvine, CA 92618-1908, USA

**Keywords:** peptides, CD13, mucin, electrochemistry

## Abstract

The adsorption of the proteins CD13, mucin and bovine serum albumin on VLGXE-Au and YNGRT-Au interfaces was monitored by electrochemical impedance spectroscopy in the presence of [Fe(CN)_6_]^3−/4−^. The hydrophobicity of the Au surface was tailored using specific peptides, blocking agents and diluents. The combination of blocking agents (ethanolamine or *n*-butylamine) and diluents (hexanethiol or 2-mercaptoethanol) was used to prepare various peptide-modified Au surfaces. Protein adsorption onto the peptide-Au surfaces modified with the combination of *n*-butylamine and hexanethiol produced a dramatic decrease in the charge transfer resistance, R_ct_, for all three proteins. In contrast, polar peptide-surfaces induced a minimal change in R_ct_ for all three proteins. Furthermore, an increase in R_ct_ was observed with CD13 (an aminopeptidase overexpressed in certain cancers) in comparison to the other proteins when the VLGXE-Au surface was modified with n-butylamine as a blocking agent. The electrochemical data indicated that protein adsorption may be modulated by tailoring the peptide sequence on Au surfaces and that blocking agents and diluents play a key role in promoting or preventing protein adsorption. The peptide-Au platform may also be used for targeting cancer biomarkers with designer peptides.

## 1. Introduction

Organized self-assembled monolayers on conductive surfaces represent a powerful approach to tuning the electrical and chemical properties of interfaces. Facile variation in properties such as conductivity, selectivity, wettability, hydrophobicity, and surface density allows for applications in material science, biosensing, bioengineering, coatings, *etc.* [1]. Moreover, adsorption of proteins at a liquid-solid interface is an important event in biomaterials and biomedical sciences [2,3,4,5]. Protein adsorption is crucial in cell adhesion for tissue regeneration, even though frequent biofouling is seen as an undesirable process in some applications. In the development of biomedical devices, understanding the protein adsorption or fouling is critical since it may compromise detection and measurement. Several key factors play a role in protein adsorption, such as multiple electrostatic, hydrogen bonding, and hydrophobic interactions, as well as ionic strength, pH, and temperature. For example, hydrophobic proteins such as human serum albumin adsorbs onto hydrophobic surfaces [6], while β-casein protein adsorbs onto hydrophilic surfaces [7]. Hence, the difference in adsorption profiles of proteins may be attributed to different protein structures, like folded globular *versus* unfolded amphiphilic conformation, among many others factors.

Despite efforts made to understand protein adsorption and achieve control over biofouling, the adsorption mechanisms for a range of proteins are still not fully understood. The adsorption mechanism for proteins including bovine serum albumin (BSA), myoglobin, fibrinogen, and lysozyme, were studied on the hydrophobic alkanethiol self-assembled monolayers using a range of techniques such as surface-plasmon resonance, quartz crystal microbalance, and field-effect transistor [8]. In addition, electrochemical methods have been used to study adsorption profiles of redox active proteins [9,10,11,12,13,14,15,16,17]. Electrochemical impedance spectroscopy (EIS) is an electrochemical method that is rapid, label-free, cost effective, and sensitive to the interfacial properties of an interface upon protein adsorption. EIS has been widely used for detection of protein/protein interactions, as well as for characterization of self-assembled monolayers and adsorption of biomolecules on surfaces [18,19,20]. The charge transfer resistance is driven by the tunneling of electrons through the surface layer or through the defects on the surface, commonly using [Fe(CN)_6_]^3−/4−^ redox probe [21]. The adsorption of protein modulated the impedance by hindering the current flow from the solution redox probe across the electrode/electrolyte interface. Decreased electron transfer rates were observed between the electrode surface and the electrolyte via protein film composed of dehydrogenase, BSA, lysozyme, *etc.* Additional electrochemical changes upon protein adsorption include the increase in impedance, decrease in current, and significant potential shift. The formation of protein layers at the electrode surface was linked to the blocking of the electron transfer kinetics. Examples of an enhanced electron transfer upon protein adsorption are less common, except for the adsorption of redox active proteins. In addition, protein adsorption onto metallic surfaces may also increase electron transfer due to corrosion of the metal [22,23].

In this work, cyclic voltammetry (CV) and EIS were used to monitor the surface properties of peptide films and protein adsorption via electron transfer perturbations on Au surfaces. The proteins of interests were CD13, mucin, and BSA CD13 is a Zn(II) dependent metalloprotease that is involved in cell proliferation, invasion and angiogenesis [24,25]. CD13 is highly expressed on myeloid cells and is a reliable biomarker for the myeloid lineage of normal and leukemic cells [26]. Mucin is a glycosylated protein that predominantly binds to the hydrophobic domain of the membranes. The over-expression of mucin has been linked to diseases such as cystic fibrosis, cancer and lung disease [27,28,29]. Hence, mucin is a potential biomarker for malignancies and other disease processes. BSA is a globular protein that is considered hydrophobic, and is commonly used in non-specific adsorption studies. Two peptides, YNGRT and VLGXE, were selected to study interaction with these proteins. These peptides have been shown to display differential binding to CD13 positive cells such as HUVEC and HT-1080 [30]. Here we demonstrated that the electron transfer of [Fe(CN)_6_]^3−/4−^ may be modulated by protein adsorption onto peptide-modified Au surfaces (VLGXE-Au and YNGRT-Au). The selection of the peptide sequence as well as the blocking agents and diluents for the surface strongly affected the impedance during protein adsorption. The enhancement or depletion of the electron transfer, due to protein adsorption, was achieved by tailoring the surface hydrophobicity and peptide content.

## 2. Experimental Section

Peptides VLGXE and YNGRT were synthesized as previously described [31]. Electrochemical experiments were performed using a CHI660D Potentiostat and gold disk electrodes (2 mm diameter, 0.031 cm^2^ surface area) (CH Instruments, Inc., Austin, TX, USA). The redox probes potassium ferricyanide(III) (K_3_[Fe(CN)_6_]) and potassium ferrocyanide(II) (K_4_[Fe(CN)_6_]) and mucin protein were purchased from Sigma-Aldrich (St Louis, MO, USA). The sodium phosphate monobasic, anhydrous, tris(hydroxymethyl)aminomethane (TRIS), and hydrogen chloride were purchased from Fisher Scientific (Waltham, MA, USA). The sodium phosphate dibasic, anhydrous as obtained from J.T. Baker (Phillipsburg, NJ, USA). CD13 protein was purchased from Novus Biologicals (Littleton, CO, USA). Potassium nitrate, bovine serum albumin and sodium hydroxide were purchased from Amresco (Cleveland, OH, USA). Glycerol was obtained from EMD (Cleveland, OH, USA) and ethanolamine from Acros Organic (Pittsburgh, PA, USA). 2-mercaptoethanol was purchased from BioRad (Hercules, CA, USA). The CD13 buffer consisted of 25 mM TRIS HCl, pH 8.0, 2% glycerol. The phosphate buffer pH 8.0 was composed of 1.7 mM sodium phosphate monobasic, and 10 mM sodium phosphate dibasic, and pH adjusted with concentrated sodium hydroxide.

### 2.1. Preparation of Peptide-Au Films

Cleaning of Au electrodes was achieved as follows: (1) etching in piranha solution for 5 min (3:1 *v*/*v* % H_2_SO_4_:H_2_O_2_); (2) hand polishing in alumina (1 μm, 0.3 μm and 0.05 μm) for 1 min each; and (3) sonicating in Milli-Q water for 5 min. Electrochemical cleaning was performed by CV in 0.5 M KOH solution at 0.5 V·s^−1^ in the −2–0 V potential range, followed by CV in 0.5 M H_2_SO_4_ at 0.5 V·s^−1^ in the 0–1.5 V potential range. The clean Au electrodes were rinsed with deionized water, and dried under N_2_ flow, and then incubated in a 2 mM solution of Lipoic acid *N*-hydroxysuccinimide ester (Lip-NHS) in ethanol for 3 days at 5 °C. Next, the Lip-NHS-modified electrodes were rinsed with ethanol. For peptide immobilization, Au electrodes were incubated with 100 μM peptide solution (YNGRT or VLGXE, 10% *v*/*v* acetonitrile in water for 24 h at 5 °C). The peptide-modified electrodes were rinsed with water and immersed in 100 mM ethanolamine solution (ethanol) for 1 h at 25 °C in order to block all unreacted NHS active ester groups. Finally, peptide-based electrodes were incubated in 10 mM 2-mercaptoethanol solution (ethanol) for 20 min at 25 °C to block any exposed Au surfaces. The electrodes were rinsed with ethanol, and dried with N_2_ gas prior to electrochemical measurements.

### 2.2. Electrochemical Measurements

Electrochemical experiments were performed using the following redox-active electrolyte: 5 mM ferri/ferrocyanide, 0.5 M potassium nitrate, 10 mM phosphate buffer, pH 8.0. CV was performed at a scan rate of 0.1 V·s^−1^ and in the potential range of −0.4 to 0.7 V, unless otherwise mentioned. EIS was carried out initially at an open circuit potential (OCP), a frequency range between 1 Hz to 100 KHz, and an amplitude of 5 mV. Experimental EIS data were fitted with an equivalent circuit using ZSimp Win 3.22 (Princeton Applied Research, Oak Ridge, TN, USA). Fitted and experimental data were presented in the form of Nyquist plots. All experiments were performed in triplicates and the corresponding error bars represent the standard deviations. The charge transfer resistance, R_ct_, was determined by fitting the impedance data to the appropriate equivalent circuit. The CV and EIS were carried out after each modification step. The peptide-based electrodes were measured before and after incubations with protein to determine the R_ct_ change.

For CD13 protein binding studies, the peptide-immobilized electrodes were incubated in the 5 µL of CD13 solution (10 µg·mL^−1^ in 25 mM TRIS·HCl, pH 8.0, 2% glycerol) for 2 h at 37 °C. The electrodes were then rinsed with 10 mM phosphate buffer, pH 8.0 and measured. The CD13 concentrations studies were carried out at 0, 0.001, 0.01, 0.1, 1, 10 and 50 µ·mL^−1^.

For experiments with BSA and mucin, the peptide-immobilized electrodes were incubated in the 5 µL of protein solution (10 µg·mL^−1^ in 25 mM TRIS·HCl, pH 8.0, 2% glycerol) for 2 h at 37 °C. Next, the electrodes were rinsed and measured. The control experiment was conducted by incubating the peptide-free electrodes in CD13 protein solution as described previously.

## 3. Results and Discussion

### 3.1. Preparation and Characterization of Peptide-Au Surfaces

**Figure 1:**
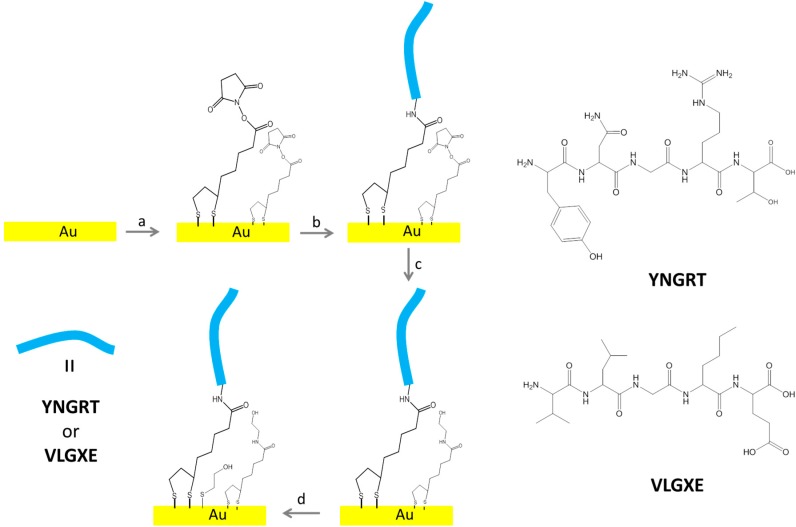
Stepwise procedure for covalent immobilization of peptide (YNGRT or VLGXE) on Au: (**a**) Lipoic acid N-hydroxysuccinimide ester (Lip-NHS); (**b**) peptide in 10% acetonitrile (YNGRT or VLGXE); (**c**) ethanolamine; (**d**) 2-mercaptoethanol.

For binding studies of proteins, two acyclic pentapeptides were chosen: YNGRT and VLGXE. The YNGRT sequence is present in the protein, fibronectin. By using conventional cell studies, the former peptide was reported to display higher binding to CD13, while the latter was not a substrate for CD13 [30]. To immobilize target peptides, clean bare Au surface was exposed to Lip-NHS solution for 3 days to ensure formation of a self-assembled monolayer as illustrated in Figure 1a.

The Lip-NHS was synthesized in house as previously described [32]. Next, the target peptide was attached via *N*-terminal to the existing disulfide monolayer as in Figure 1b. Amide conjugation chemistry was used to prepare YNGRT-Au and VLGXE-Au surfaces. The VLGXE peptide has a single reactive amine group at the *N*-terminal which is presumably used for amide conjugation leading to a single VLGXE peptide orientation on the surface. The YNGRT peptide is overall positively charged and contains two basic functional groups, the *N*-terminal amine and guanidine in Arginine (R) side chains. The *N*-terminal, amine and protonated guanidinium of the R group are expected to have pKa values of 9 and 12, respectively. With the *N*-terminal amine being the most reactive, coupling is expected via the *N*-terminal. In addition, YNGRT is overall positively charged while VLGXE is negatively charged. At a working pH this may lead to different non-specific electrostatic interactions with the Au surface for YNGRT peptide during the immobilization step. Recent studies have identified Arginine as the charged amino acid that facilitates initial binding to Au surface [33]. Hence, the Arginine residue in YNGRT may bind electrostatically to the exposed Au sites. To block unreacted NHS sites, the blocking agent, ethanolamine, was used (Figure 1c). The backfilling of any exposed Au surface was achieved by using the diluent, 2-mercaptoethanol (Figure 1d), which also minimized any non-specific protein adsorption due to hydrophobic interactions. The YNGRT-Au and VLGXE-Au surfaces were characterized using the electrochemical methods.

**Figure 2:**
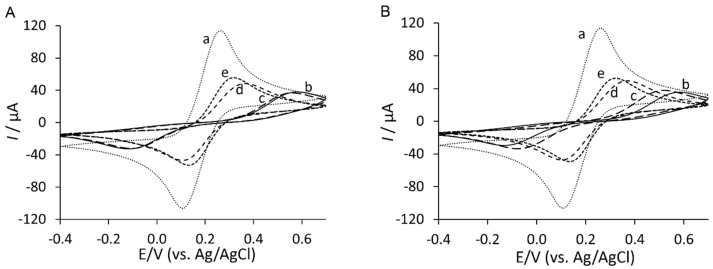
Cyclic voltammograms of the step-wise surface fabrication of (**A**) YNGRT-Au and (**B**) VLGXE-Au: (a) bare Au; (b) Lip-NHS; (c) peptide; (d) ethanolamine; and (e) 2-mercaptoethanol.

The surface modification steps were monitored by cyclic voltammetry and electrochemical impedance spectroscopy in the presence of [Fe(CN)_6_]^3−/4−^. The cyclic voltammograms of stepwise surface preparation of YNGRT-Au and VLGXE-Au are presented in Figure 2A,B, respectively.

Compared to the bare Au electrode (a), the immobilization of Lip-NHS (b) produced a decrease in current and an increase in the potential shift. Then the immobilization of the peptides was characterized by a reduced current. The YNGRT immobilization produced a lower current and greater potential separation than the VLGXE immobilization, presumably due to the numerous points of attachment, namely an *N*-terminal amine for covalent attachment and an Arginine side chain for non-covalent binding to the Au surface. The subsequent blocking by ethanolamine (d) and backfilling with 2-mercaptoethanol (e) produced the final YNGRT-Au and VLGXE-Au surfaces.

**Figure 3:**
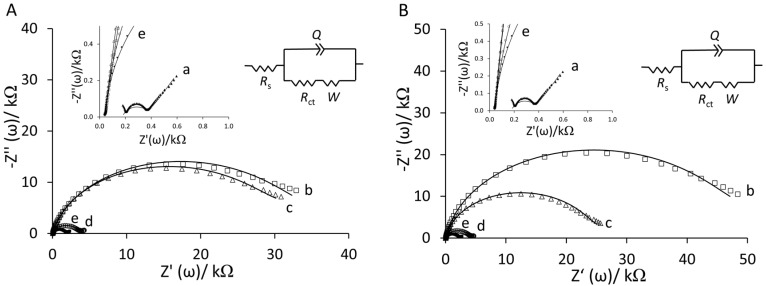
Nyquist plots of the step-wise surface fabrication of (**A**) YNGRT-Au and (**B**) VLGXE-Au: (a) bare Au, (b) Lip-NHS, (c) peptide, (d) ethanolamine and (e) 2-mercaptoethanol (inset: equivalent circuit used for fitting impedance data).

The stepwise surface modification during fabrication of YNGRT-Au and VLGXE-Au was also monitored by EIS (Figure 3).

**Figure 4:**
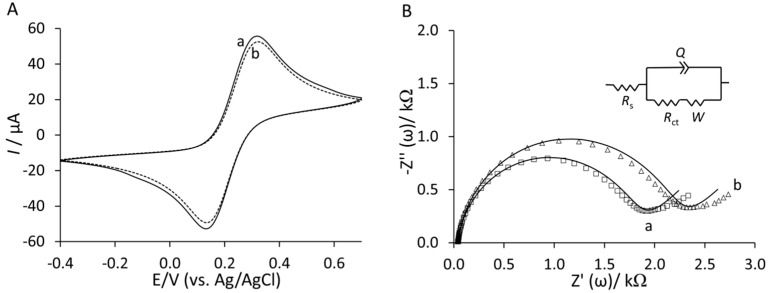
(**A**) Cyclic voltammograms and (**B**) Nyquist plots of YNGRT-Au (a) and VLGXE-Au (b) surfaces.

Nyquist plots in Figure 3A,B show that Lip-NHS (b) produced higher resistance and a larger semi-circle portion of the curve. Subsequent immobilization of peptides (c) produced lower resistance presumably due to introduction of charge from peptide films. Interestingly, the blocking with ethanolamine (d) produced much lower impedance due to replacement of some non-specifically adsorbed peptides on the surface. The final backfilling step with 2-mercaptoethanol (e) produced a slightly lower resistance. The CVs and Nyquist plots of the final YNGRT-Au (a) and VLGXE-Au (b) surfaces are presented in Figure 4. CVs for both peptide surfaces were similar (Figure 4A). The semi-circle in the Nyquist plots indicated significant charge-transfer resistance, R_ct_, due to immobilization of peptide films on Au electrodes. The impedance was fitted to the equivalent circuit (Figure 4B inset) and the impedance fitted parameters are presented in Table 1. The equivalent circuit was composed of solution resistance, R_s_, in series with the complex component, the constant phase element, CPE (Q), in parallel with the charge transfer resistance, R_ct_, and Warburg constant, W.

**Table 1:** Impedance parameters fitted for YNGRT-Au and VLGXE-Au surfaces using the equivalent circuit in Figure 4B (inset).

	R_S_ (kΩ)	CPE (µΩ^−1^·s^n^)	*n*	R_ct_ (kΩ)	W (µΩ^−1^·s^0.5^)
YNGRT-Au	0.04 ± 0.01	1.10 ± 0.13	0.94 ± 0.01	2.46 ± 0.69	523 ± 86.2
VLGXE-Au	0.04 ± 0.01	1.33 ± 0.23	0.93 ± 0.01	1.82 ± 0.35	570 ± 88.9

### 3.2. Protein Binding to Peptide-Au Surfaces

**Figure 5:**
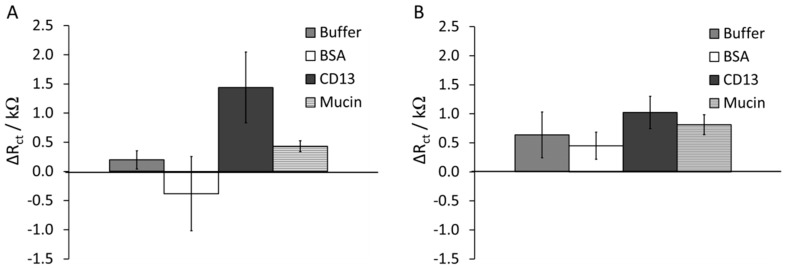
Plot of change in R_ct_ as a function of protein solution during adsorption onto YNGRT-Au (**A**) and VLGXE-Au (**B**) surfaces (each protein was at 10 µg·mL^−1^ whereas buffer refers to the protein-free solution).

Adsorption of proteins was studied at open circuit potential and in 5 mM [Fe(CN)_6_]^3−/4−^, 0.5 M potassium nitrate, and 10 mM phosphate buffer, pH 8.5. The peptide-modified Au surfaces were exposed to various protein solutions for 2 h at 37 °C in order to induce protein adsorption and binding. The proteins under investigation were CD13, mucin, and BSA. In the event of favorable protein binding to the immobilized peptide, the impedance was expected to change. After incubation in 10 µg·mL^−1^ of protein, the peptide-modified electrodes were rinsed and measured by EIS. The plots of change in R_ct_ as a function of protein for YNGRT-Au and VLGXE-Au surfaces are depicted in Figure 5.

The binding of CD13 to YNGRT-Au increased the R_ct_ value by 1.5 ± 0.42 kΩ (Figure 5A). The R_ct_ values for YNGRT-Au and CD13-YNGRT-Au were 0.48 ± 0.42 kΩ and 1.91 ± 0.97 kΩ, respectively. In contrast, in the absence of protein (buffer), very little change in R_ct_ was observed. Notably, BSA resulted in a 25% decrease in impedance (negative ΔR_ct_ value). Also, there was an increase in impedance in the presence of mucin, but much less so than for CD13.

An increase in impedance was observed when VLGXE-Au was exposed to all three proteins (Figure 5B). The R_ct_ values for VLGXE-Au and CD13-VLGXE-Au were 0.68 ± 0.19 kΩ and 1.02 ± 0.28 kΩ, respectively. The BSA and mucin binding produced large ΔR_ct_ values as well. Notably, a dramatic change in impedance in the presence of buffer (protein free solution) suggests some rearrangement of peptide film. Previous studies also reported slight binding of this sequence to CD13 protein overexpressed on the HUVEC or HT-1080 cell lines [30]. Notably, even mucin binding to VLGXE-Au surface produced an increase in electrochemical signal.

At an adsorption solution pH and electrolyte pH of 8.0, it is expected that all three proteins studied contain an overall negative charge. BSA is a relatively small protein (66 kDa molecular weight, 14 nm × 4 nm size), highly hydrophobic and pI ~ 5. Hence when at pH 8.0, it is expected that BSA is overall negatively charged. Mucin is a large protein (120–225 kDa) existing as a random coil around pH 7, with an end-to-end length of 390 nm and persistence length of 8–10 nm [34]. The isoelectric point of mucin has been reported to be between 2 and 3. At pH 8.0, mucin is not expected to undergo any aggregation or gelation, since it presumably exists as a monomer and is overall negatively charged [35]. At pH 8.0, mucin may exist as an extended protein in a random coil conformation. The CD13 protein has a molecular mass of 150 kDa and pI of 4 [36].

From Figure 5A it is apparent that CD13 binds and adsorbs to YNGRT-Au specifically and caused the greatest increase in the surface coverage, *i.e.*, an increase in charge transfer resistance. This increase indicates that YNGRT-Au binds CD13 specifically. Despite the overall negative charge of all three proteins, CD13 caused the greatest increase in the electrochemical signal, indicating that electrostatic interactions are not the main cause of the electrochemical signal modulations. Since CD13 and mucin are of similar molecular weights (almost double that of BSA), the dramatic signal increase with CD13 may not be attributed to the overall size of the protein, but rather to a specific interactions between CD13 and YNGRT peptide, which is lacking in the case of mucin. Overall, the results (Figure 5) highlight that the YNGRT-Au surface binds selectively to CD13, whereas the VLGXE-Au surface is not selective and binds to all three proteins to somewhat similar extent.

### 3.3. Effects of Surface Composition on Protein Adsorption

To understand protein binding and adsorption to the peptide-Au surface, various interfaces were prepared by changing the blocking agent/diluent combination: (a) ethanolamine/2-mercaptoethanol; (b) ethanolamine/hexanethiol; (c) *n*-butylamine/hexanethiol; and (d) *n*-butylamine/2-mercaptoethanol. The ethanolamine/2-mercaptoethanol-treated surfaces may be considered highly hydrophilic. The protein binding to this type of surface was described in Section 3.2. The ethanolamine/hexanethiol and *n*-butylamine/2-mercaptoethanol peptide-Au surfaces were considered partially hydrophilic. By contrast, *n*-butylamine/hexanethiol peptide-Au surface may be considered highly hydrophobic. The ranking of hydrophobicity is summarized in Table 2.

**Table 2:** Summary of combinations of blocking agent and diluent with corresponding hydrophobicity.

Combination	Blocking Agent	Diluent	Hydrophobicity
a	Ethanolamine	2-mercaptoethanol	Highly hydrophilic
b	Ethanolamine	hexanethiol	Partially hydrophilic
c	*n*-butylamine	hexanethiol	Highly hydrophobic
d	*n*-butylamine	2-mercaptoethanol	Partially hydrophobic

**Figure 6:**
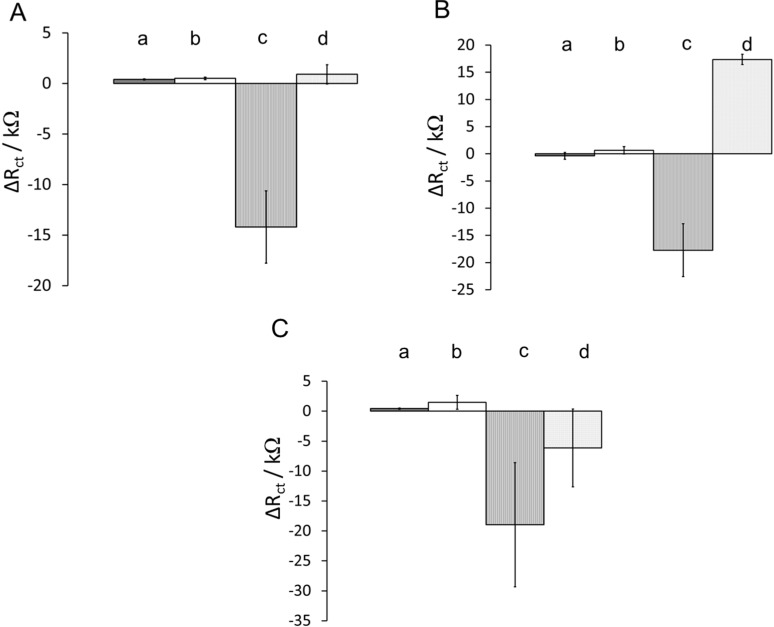
Plot of change in R_ct_ as a function of protein solution during (**A**) CD13; (**B**) BSA; and (**C**) mucin protein adsorption on YNGRT-Au with various surfaces: (a) ethanolamine/2-mercaptoethanol, (b) ethanolamine/hexanethiol, (c) *n*-butylamine/hexanethiol, (d) *n*-butylamine/2-mercaptoethanol (protein concentration was 10 µg·mL^−1^).

Figure 6A depicts binding of CD13 protein to various YNGRT-Au surfaces with different hydrophobicity. The highly nonpolar surface (c) produced a large decrease in ΔR_ct_ of −14 ± 3.5 kΩ upon adsorption of CD13. However, other partially hydrophilic and highly hydrophilic surfaces produced relatively small increases in ΔR_ct_ of ~1 kΩ. Similarly, BSA (Figure 6B) and mucin (Figure 6C) adsorptions produced large ΔR_ct_ values when highly nonpolar blocking agent and diluent were used (c). Hence, a significant electrochemical signal change may be due to largely hydrophobic interactions during protein binding or adsorption to YNGRT-Au surfaces. The hydrophobic proteins are expected to adsorb onto hydrophobic surfaces more so than onto hydrophilic surfaces [37]. The electrochemical data suggest that all three proteins have similar hydrophobic content and all adsorb onto largely hydrophobic surfaces.

**Figure 7:**
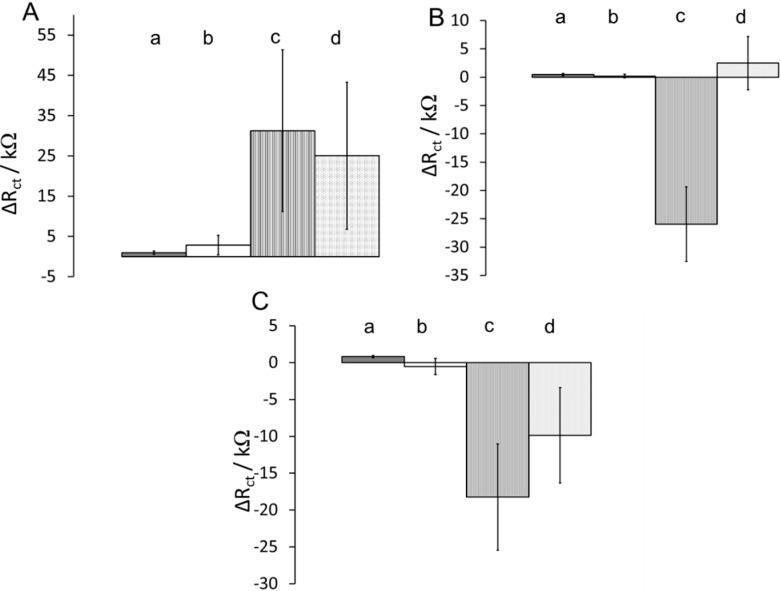
Plot of change in R_ct_ as a function of protein solution during (**A**) CD13; (**B**) BSA; and (**C**) mucin protein adsorption on VLGXE-Au with various surfaces: (a) ethanolamine/2-mercaptoethanol, (b) ethanolamine/hexanethiol, (c) n-butylamine/hexanethiol, (d) n-butylamine/2-mercaptoethanol (protein concentration was 10 µg·mL^−1^).

When the VLGXE-Au interface was prepared with the various combinations of blocking agents and diluents, the protein adsorption and binding was also measured by impedance. When ethanolamine/2-mercaptoethanol and ethanolamine/hexanethiol were used in preparation of the VLGXE-Au surface, the adsorption of CD13, BSA and mucin produced similar responses with low values of ΔR_ct_. The large increase in ΔR_ct_ was observed with the n-butylamine blocking agent (c and d). In Figure 7B, BSA adsorption onto VLGXE-Au surface with n-buthylamine/hexanethiol (c) produced the largest ΔR_ct_ of −25 ± 6.5 kΩ. This may be indicative of the largest BSA adsorption onto this surface compared to others. All other surfaces yielded a small increase in ΔR_ct_ for BSA. The adsorption of BSA onto bare-Au, cysteine-Au and thiophenol-Au surfaces was reported at pH 7.3, and BSA adsorption was positively correlated to the surface hydrophilicity of the electrode [38].

The adsorption of the mucin protein onto various VLGXE-Au surfaces was also monitored (Figure 7C). The adsorption onto highly nonpolar surface containing n-butylamine/hexanethiol (c) produced the largest decrease in ΔR_ct_ at −18 ± 7.2 kΩ, as was seen for other proteins. Interestingly, the partially hydrophilic surface (d) (n-butylamine/2-mercaptoethanol) also produced a decrease in ΔR_ct_ of −9.8 ± 6.5 kΩ. This was not observed for BSA.

At pH 8.0, the YNGRT-Au interface was overall neutral, but the VLGXE-Au interface may be overall negatively charged. Hence, the negatively charged proteins should be less attracted to the negatively charged peptide film, *i.e.*, VLGXE-Au. However, it seems that the VLGXE-Au surface with hydrophobic blocking agent and diluent induced the largest protein adsorption.

The electrochemical data indicated that highly nonpolar surfaces induced the greatest change in charge transfer resistance for all three negatively charged proteins under investigation. This trend suggested significant protein adsorption onto highly nonpolar surfaces, based on the blocking agent, n-butylamine, which may be driven via hydrophobic rather than electrostatic interactions. Importantly, the introduction of hydrophilic blocking agent or diluent significantly reduced protein adsorption or binding and had little effect on the R_ct_. The decrease in R_ct_ values, following protein binding, may be due to protein reorientation on the surface and formation of holes for a more facile electron transfer from the solution redox probe to the electrode. While other processes may be occurring during adsorption, such as hydrogen bonding, electrostatic and Van der Waals interactions, the hydrophobic interactions seemed to dominate the electrochemical trend. In addition, applied potential during electrochemical measurement may influence the protein adsorption. All measurements were carried out at open circuit potential, where peptide films may be neutral or negatively charged. Since both peptide films produced a similar electrochemical response, the proteins may adsorb to a similar extent on both peptide surfaces. However, the level of surface hydrophobicity rather than electrostatic interactions predominately contributed to protein adsorption.

## 4. Conclusions/Outlook

The electrochemical detection of proteins on immobilized peptide-Au platforms was demonstrated. The surface modification dramatically influenced protein adsorption, indicating that in the design of peptide-based biosensors, careful selection of blocking agent and diluent is required. Overall, highly hydrophobic surface induced the greater electrochemical response, suggesting maximum protein adsorption. The peptide-based platform described here may be used with the custom peptides library to develop a multiplexed assay for detection of multiple cancer biomarkers in a single device. This application would allow for point-of-care diagnosis and drug and disease progression screening.
